# Socioeconomic and immigration status and COVID-19 testing in Toronto, Ontario: retrospective cross-sectional study

**DOI:** 10.1186/s12889-022-13388-2

**Published:** 2022-05-29

**Authors:** Braden O’Neill, Sumeet Kalia, Susan Hum, Peter Gill, Michelle Greiver, Abirami Kirubarajan, David Eisen, Jacob Ferguson, Sheila Dunn

**Affiliations:** 1 MAP Centre for Urban Health Solutions, Li Ka Shing Knowledge Institute, St. Michael’s Hospital, Unity Health Toronto, Toronto, Canada; 2 Department of Family and Community Medicine, St. Michael’s Hospital, Unity Health Toronto, Toronto, Canada; 3 Department of Family and Community Medicine, Faculty of Medicine, University of Toronto, Toronto, Canada; 4 Department of Family and Community Medicine, Women’s College Hospital, Toronto, Canada; 5 Women’s College Research Institute, Women’s College Hospital, Toronto, Canada; 6 Institute for Health Policy, Management and Evaluation, University of Toronto, Toronto, Canada; 7 Department of Pediatrics, University of Toronto, Toronto, Canada; 8 Pediatric Outcomes Research Team, Division of Pediatric Medicine, Department of Pediatrics, The Hospital for Sick Children, Toronto, Canada; 9 Department of Family and Community Medicine, North York General Hospital, Toronto, Canada; 10 Undergraduate Medical Education, Temerty Faculty of Medicine, University of Toronto, Toronto, Canada

**Keywords:** COVID-19, Social determinants of health, Primary health care, Immigration, Diagnostic tests and procedures

## Abstract

**Background:**

Preliminary evidence suggests that individuals living in lower income neighbourhoods are at higher risk of COVID-19 infection. The relationship between sociodemographic characteristics and COVID-19 risk warrants further study.

**Methods:**

We explored the association between COVID-19 test positivity and patients’ socio-demographic variables, using neighborhood sociodemographic data collected retrospectively from two COVID-19 Assessment Centres in Toronto, ON.

**Results:**

Eighty-three thousand four hundred forty three COVID-19 tests completed between April 5–September 30, 2020, were analyzed. Individuals living in neighbourhoods with the lowest income or highest concentration of immigrants were 3.4 (95% CI: 2.7 to 4.9) and 2.5 (95% CI: 1.8 to 3.7) times more likely to test positive for COVID-19 than those in highest income or lowest immigrant neighbourhoods, respectively. Testing was higher among individuals from higher income neighbourhoods, at lowest COVID-19 risk, compared with those from low-income neighbourhoods.

**Conclusions:**

Targeted efforts are needed to improve testing availability in high-risk regions. These same strategies may also ensure equitable COVID-19 vaccine delivery.

**Supplementary Information:**

The online version contains supplementary material available at 10.1186/s12889-022-13388-2.

## Introduction

It has been over 2 years since a cluster of ‘atypical pneumonia’ was reported in Wuhan City, Hubei Province, China [1, 2]. Since then, COVID-19 has spread worldwide, resulting in unprecedented upheaval of social, economic, and political systems. By March 2022, there have been 455 million reported cases of COVID-19, with 6 million deaths [3].

“Test and trace” approaches, involving different types of tests and strategies for determining testing eligibility, rely on identifying, testing, contact tracing and isolating infected individuals. These approaches have formed a core component of the pandemic response across Canada [4].

As part of the initial response to COVID-19, the Ontario government developed ‘COVID-19 Assessment Centres’ testing individuals for SARS-CoV-2 with nasal or nasopharyngeal swabs, analysed using polymerase chain reaction (PCR) technology [5]. By March 31, 2020, Toronto Ontario – Canada’s largest city with a population of 3 million people within a metropolitan area of about 6 million [6] – had opened 11 COVID-19 Assessment Centres [7]. Testing eligibility and manner of testing (i.e., by walk-in or by appointment only) varied over time and by assessment centres. Initially, testing focused mainly on high risk individuals (e.g. symptoms consistent with COVID-19) but over time, testing criteria changed (Table 1) to include asymptomatic people who were ‘concerned’ about an infection and those who were visiting congregate living settings (e.g. long-term care facilities). The capacity to conduct diagnostic testing has limits, and over-utilization of this resource can lead to reporting delays and unnecessary costs. To optimize its utility, diagnostic testing should be readily available to those who are most likely to benefit from it, identifying cases among people who are likely to have COVID-19 infection, and/or transmit it to others.

**Table 1:** Timeline of key COVID assessment centre announcements in Ontario during study period

Date	Announcement	Reference
March 12/20	COVID assessment centres opened at six locations in Ontario	[7]
May 24/20:	Premier recommended ‘everyone gets a test’	[8]
Sept 23/20:	Asymptomatic people could access tests at private pharmacies	[9]
Sept 24/20	Testing at assessment centres restricted to those with symptoms of COVID or close contacts or who are at high risk	[10]
Oct 2/20	All Ontario COVID assessment centres switched to ‘by appointment only’	[11]

In May 2020, a study from Ontario’s Institute for Clinical and Evaluative Sciences (ICES) using provincial administrative health data reported higher rates of SARS-CoV-2 test positivity and hospitalization among people living in geographical areas associated with lower socioeconomic status, as well as areas with higher proportions of immigrants and ethnic minorities, as compared to those living in higher income neighbourhoods [12]. These findings were consistent with studies in other jurisdictions that identified low socioeconomic status and immigrant status as determinants of higher risk for COVID-19 infection and poorer outcomes from the disease [13, 14]. The relationship between socioeconomic or immigrant status and COVID-19 infection may in part be mediated by occupational risk factors. Residents in low-income neighbourhoods and immigrants are disproportionately likely to work in sectors with high exposure risk to COVID-19, such as healthcare or other essential workplaces that remain open during the pandemic [13, 14]. These individuals may also be at higher risk due to multi-generational homes with a higher proportion of older adults, and may also face barriers in accessing personal protective equipment due to prohibitive costs or language barriers, as well as barriers in accessing testing such as getting time off work, transportation to testing sites [15]. Understanding these patient-level demographic factors are essential for identifying who is at highest risk of COVID-19 and this knowledge can be used to guide allocation of limited resources such as testing. The purpose of this study was initially to inform local resource allocation at two Toronto COVID-19 assessment centres and their corresponding hospitals. These results build on what has been completed by ICES, describing the association between SARS-CoV-2 test positivity and patient socio-demographic characteristics specifically at the level of service delivery at assessment centres.

## Methods

### Study design and setting

We conducted a retrospective cohort study using data collected at two COVID-19 Assessment Centres in Toronto, Ontario: one at a medium-sized community hospital 16 km north of downtown Toronto (North York General Hospital; NYGH) and the second at an academic, ambulatory care hospital in downtown Toronto, with a focus on women’s health (Women’s College Hospital; WCH). The population of Toronto is about 50% immigrants, with 85% having arrived before 2011 [16]; the average household income in Toronto is $98,174 [17]. There has been increasing income inequality in Toronto in the last several decades; the two study settings here differ in that the area around NYGH has a ‘very high’ income level (140–697% of the average income in 2012) and the area around WCH has a ‘very low’ income level (36–60%) [18]. Patients did not have to pay for COVID-19 testing. Our study was approved by NYGH’s and WCH’s Research Ethics Boards (Protocols 20–0021 and 2020–0059-E, respectively).

### Data collection

We extracted routinely collected data from these two centres between April 5 – September 30, 2020, including patient age, sex, confirmed or suspected close contact with an infected individual, and SARS-CoV-2 test results. All SARS-CoV-2 diagnostic tests completed were polymerase chain reaction (PCR) assays. Residential postal codes were collected and used to identify neighbourhood level income quintiles and immigration terciles, using Statistics Canada postal code conversion files [19, 20]. Immigration data used in this study represent proportion of residents in an area who were born outside Canada. For all extracted data elements, the initial data collection was performed by individuals not involved with the study. The majority of data elements were either objective details obtained from patient-level electronic health records (e.g., patient age, sex) or were extracted from official government records (e.g., residential postal codes). This retrospective chart review used data collected during routine clinical care which were deidentified prior to analysis. Data were collected, handled, and analyzed in accordance with local data protection regulations.

During the study period, the results of all SARS-CoV-2 tests conducted at the two sites were included for analysis, except subsequent ones conducted after an initial positive test. This avoided duplicate data, as it would not be a new infection. Early in the study period, Public Health Ontario recommended ‘re-testing’ to assess disease resolution, but this practice changed after it became clear that ‘repeat positive’ tests reflected inactive viral shedding, rather than ongoing clinical disease [21]. Our methodology has been described in greater detail in another manuscript which characterized the predictive value of ‘anosmia’ as an early symptom of COVID-19 infection, and described the clinical presentation of patients who tested positive for SARS-CoV-2 [22].

### Data analysis

Descriptive statistics were used to describe the study population and test characteristics. We then assessed the linear trend in positivity rate of COVID-19 swab test with respect to income quintiles and immigration terciles using the two-sided Cochrane-Armitage test with 0.05 significance level [23]. Further, we used generalized estimating equations [24] to assess the odds of a positive COVID-19 test across the geographical regions related to neighbourhood level income quintile and immigration tercile. There is substantial heterogeneity in income within postal codes, and there are several available approaches to optimize the representation of income from postal code data. The first possible approach would be to use the Postal Code Conversion File (PCCF) single linkage indicator (or matching criteria) to identify the majority of the dwellings assigned to a particular postal code [25]. This approach introduces bias because it ignores smaller dwellings which typically have lower socio-economic status. PCCF+ uses population-weighted random allocation for postal codes that link to more than one dissemination area [26].) Since each postal code may span multiple dissemination areas (or blocks), we chose this approach to optimize adequate representation of the entire population living in each postal code. We did not conduct an a priori sample size calculation; the sample sizes were sufficiently large (> 83,000 tests) to ensure sufficient power (> 80%) even with small effect sizes. Odds ratios were adjusted for age, sex, and travel history [and recorded symptoms/signs (cough and/or shortness of breath, fever, anosmia, diarrhea and/or abdominal pain, temperature and pulse rate)]. We used complete-case analysis for hypothesis testing using the Cochrane-Armitage test and generalized estimating equations, because we assessed that missing data were not at random. We pooled data from both institutions prior to analysis. Analyses were performed using SAS version 9.4 (SAS Corp.).

## Results

Characteristics of patients who had SARS-CoV-2 tests at either site are described in Table 2. In total, the two sites completed 83,443 tests (53,479 at NYGH and 29,964 at WCH) (Table 3). The positivity rate at NYGH was 2.26, and 1.45% at WCH. There were 1817 total positive tests across both sites; 177 were excluded for being ‘repeat positives’, leaving 1640 positive incident cases of COVID-19 across both sites. A higher positivity rate was observed among adults aged 40–49 years (2.57%) as compared to youth (0–9 years, 1.00%; 10–19 years, 1.89%) and older adults (60–69, 1.61%; 70+, 0.93%) (Table 3). COVID-19 test positivity was more than three times higher among people who lived in the lowest-income versus highest-income neighbourhoods (3.83% vs. 1.11%), and in regions with the highest versus lowest proportion of immigrants (2.82% vs. 0.89%). According to the Public Health Ontario’s COVID-19 Data Tool, the average test positivity rate in Ontario from April 5th 2020 to September 30th 2020 was 1.57% [27]. There were < 1% missing data for every included variable (Table 1).

**Table 2:** Participant characteristics with respect to NYGH^a^ and WCH^b^ sites

*Characteristics*	*Site*				*Total*
	*NYGH*^a^		*WCH*^b^		
	*N*	*Column Percent (%)*	*N*	*Column Percent (%)*	*N*
*Age group (years)*					
*0–9 years*	3108	5.81%	1501	5.01%	4609
*10–19 years*	4072	7.61%	1119	3.73%	5191
*20–29 years*	10,770	20.14%	8143	27.18%	18,913
*30–39 years*	8987	16.80%	7927	26.46%	16,914
*40–49 years*	8174	15.28%	3749	12.51%	11,923
*50–59 years*	8353	15.62%	3355	11.20%	11,708
*60–69 years*	6281	11.74%	2429	8.11%	8710
*> 70 years*	3729	6.97%	1741	5.81%	5470
*Missing*	5	0.01%	0	0%	5
*Sex*					
*Female*	29,470	55.11%	19,094	63.72%	48,564
*Male*	24,009	44.89%	10,870	36.28%	34,879
*Income Quintiles*					
*1(=lowest)*	11,065	20.69%	4455	14.87%	15,520
*2*	9866	18.45%	5155	17.20%	15,021
*3*	8910	16.66%	4836	16.14%	13,746
*4*	9691	18.12%	4668	15.58%	14,359
*5*	13,850	25.90%	10,019	33.44%	23,869
*Missing*	97	0.18%	831	2.77%	928
*Immigration terciles*					
*1(=lowest foreign-born population)*	6503	12.16%	5469	18.25%	11,972
*2*	17,930	33.53%	16,860	56.27%	34,790
*3*	28,903	54.05%	6552	21.87%	35,455
*Missing*	143	0.27%	1083	3.61%	1226
*Travel*					
*No*	52,619	98.39%	29,057	96.97%	81,676
*Yes*	860	1.61%	907	3.03%	1767
*Total*	53,479	100.00%	29,964	100.00%	83,443

^a^*NYGH* North York General Hospital, ^b^*WCH* Women’s College Hospital

**Table 3:** Patient demographics with respect to positive or negative COVID-19 swab test

*Characteristics*	*COVID-19 swab test*			*Total*
	*Negative*	*Positive*		
	*N*	*N*	*Prevalence (%)*	*N*
*Site*				
NYGH*(site 1)	52,272	1207	2.26%	53,479
WCH** (site 2)	29,531	433	1.45%	29,964
*Age group (years)*				
*0–9 years*	4563	46	1.00%	4609
*10–19 years*	5093	98	1.89%	5191
*20–29 years*	18,488	425	2.25%	18,913
*30–39 years*	16,602	312	1.84%	16,914
*40–49 years*	11,617	306	2.57%	11,923
*50–59 years*	11,446	262	2.24%	11,708
*60–69 years*	8570	140	1.61%	8710
*70+ years*	5419	51	0.93%	5470
*Missing*	5	0	0%	5
*Sex*				
*Female*	47,646	918	1.89%	48,564
*Male*	34,157	722	2.07%	34,879
*Income Quintiles*				
*1(=lowest)*	14,926	594	3.83%	15,520
*2*	14,699	322	2.14%	15,021
*3*	13,523	223	1.62%	13,746
*4*	14,130	229	1.59%	14,359
*5*	23,603	266	1.11%	23,869
*Missing*	922	6	0.65%	928
*Immigration terciles*				
*1(=lowest foreign-born population)*	11,866	106	0.89%	11,972
*2*	34,268	522	1.50%	34,790
*3*	34,454	1001	2.82%	35,455
*Missing*	1215	11	0.90%	1226
*Travel*				
*No*	80,090	1586	1.94%	81,676
*Yes*	1713	54	3.06%	1767
*Total*	81,803	1640	1.97%	83,443

*NYGH North York General Hospital, **WCH Women’s College Hospital

**Fig. 1:**
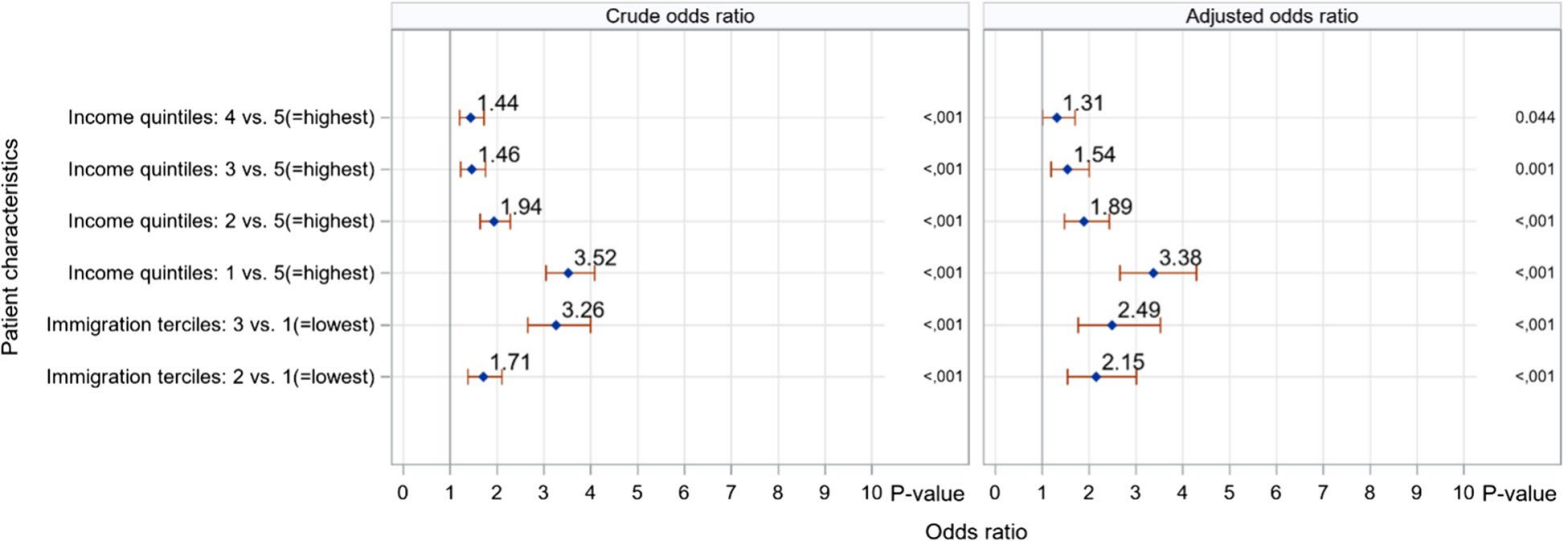
Odds ratios for positive COVID-19 swab test by income quintile and immigration tercile

Figure 1 shows the odds ratios of a positive test related to neighbourhood income quintile and immigration tercile. (The numerical values are available in Table S1.) After adjustment for potential confounding variables, individuals living in neighbourhoods with the lowest versus highest income quintile were 3.38 times more likely to test positive for SARS-CoV-2 (95% CI: 2.66 to 4.29). In addition, those living in neighbourhoods with the highest versus lowest immigrant population were 2.49 times more likely to test positive (95% CI: 1.77 to 3.52).

Table 4 shows the positivity rate of SARS-CoV-2 in different income quintiles, within each immigration tercile. With the exception of areas in the lowest immigrant tercile (i.e., lowest foreign-born population), SARS-CoV-2 positivity rates increased as neighbourhood-level income decreased. The highest positivity rate (4.14%; 517/12486) was in neighbourhoods with the lowest income and highest immigrant population. Regarding testing volume, Table 4 shows that individuals at higher risk for COVID-19 infection generally received less testing. Particularly, in the middle immigrant tercile, income quintiles with higher SARS-CoV-2 positivity rates had a lower total number of tests. In the lowest immigrant tercile, four income quintiles similarly have an inverse relationship between test positivity and number of tests. The highest immigrant tercile is an exception to this trend, as income quintiles with higher positivity rates also had a higher testing volume.

**Table 4:** SARS-CoV-2 test positivity by demographic characteristics

*Characteristics*		*COVID-19 swab test*				*Total*
		*Negative*	*Positive*			
		*N*	*N*	*Prevalence (%)*	*P-value* †	*N*
*Immigration terciles*	*Income Quintiles*	337	4	1.17%	0.34	341
*1(=lowest foreign-born population)*	*1(=lowest)*					
	*2*	703	6	0.85%		709
	*3*	892	10	1.11%		902
	*4*	1659	18	1.07%		1677
	*5(=highest)*	8262	68	0.82%		8330
*2*	*1(=lowest)*	2566	70	2.66%	< 0.0001	2636
	*2*	4776	75	1.55%		4851
	*3*	6256	97	1.53%		6353
	*4*	7686	122	1.56%		7808
	*5(=highest)*	12,984	158	1.20%		13,142
*3(=highest)*	*1(=lowest)*	11,969	517	4.14%	< 0.0001	12,486
	*2*	9118	240	2.56%		9358
	*3*	6252	115	1.81%		6367
	*4*	4759	89	1.84%		4848
	*5(=highest)*	2356	40	1.67%		2396
*Total*		80,575	1629	1.98%		82,204

† *P*-value assesses the existence of a linear trend in increasing positivity rate of COVID-19 swab test with decreasing income quintiles for each immigration tercile

*N* = 82,204 in this table (*N* = 83,443 in Tables 1, 2, and 3) because missing data for immigration and income were removed prior to the analysis presented here

Table S2 displays the positivity rate in different immigrant terciles, within each income quintile, and found that within every income quintile the SARS-CoV-2 positivity rate increased as neighbourhood immigration tercile increased. There was no clear relationship between testing volume and the positivity rate of different immigration terciles, within each income quintile.

## Discussion

In this retrospective chart review study using neighborhood socioeconomic data collected from two Toronto-area COVID-19 Assessment Centres, neighbourhood income and immigration levels were associated with COVID-19 test positivity but in opposing directions. People living in the lowest income and highest immigrant neighbourhoods were more likely to test positive than those from higher income neighbourhoods or lower immigrant neighbourhoods. However, individuals living in higher risk communities were generally less likely to be tested than those living in more affluent communities, where the risk of contracting COVID-19 was lower.

Our findings of associations for test positivity are consistent with initial Ontario-wide studies [12, 28] and from other studies across multiple jurisdictions [29, 30]. The finding of higher COVID-19 test positivity among individuals living in neighbourhoods with higher proportions of immigrants and newcomers has also been reported elsewhere, such as in New York City [31], and Montreal, Quebec [32]. These findings have been attributed to a higher proportion of essential workers, who often live in low income, marginalized neighbourhoods, and work in higher risk occupational settings, (e.g., long-term care centres, food processing plants, warehouses and factories) [33, 34], where public health measures may be lax.

Low income and immigration are often associated with poorer access to health care. In our research, only low income was associated with decreased testing, while people living in high immigrant neighbourhoods were more likely to be tested. Findings from other studies on testing and income are conflicting. A New York City study found no association [31], whereas a survey of US adults in 2020 found that lower income was associated with perceived difficulty with accessing testing [30]. Our finding of increased testing volume in high immigrant neighbourhoods contrasts with an Ontario provincial report which found that immigrants overall had lower COVID-19 test rates than the general population, with an exception for the subgroup of immigrants employed as economic caregivers [12]. It is possible that our findings reflect the catchment area of the two assessment centres. WCH is located in the ‘Bay Street Corridor’ neighbourhood which is more densely populated than the ‘Westminster-Branson’ neighbourhood where the NYGH is located (14,000 people/km^2^ vs 7000/ km^2^) but both have similar total populations (25,000 vs 26,000, respectively). The Bay Street Corridor neighbourhood has lower recent immigrant population (41% vs 70%); higher proportion of people living in poverty (39% vs 27.2%); more renters (79% vs 60%); more people living in higher-density housing such as apartments (98% vs 76%); and an overall higher education level (> undergraduate university degree 79% vs 44%) [35]. However, anyone could seek testing at the centres and people testing did not necessarily live near them.

Many testing approaches to limit the spread of COVID-19 have been suggested in the literature, including testing everyone in a population either once [36] or recurrently, such as weekly [37]. Rapid tests have also been recommended, particularly for higher-incidence settings such as high-risk work-places, but their uptake in Canada has been limited [38]. The optimal strategy is not known and is affected by local outbreak conditions and system capacity for testing. There are inevitable limitations in system capacity with widespread population testing, as demonstrated by the ‘backlog’ of almost 100,000 collected samples waiting to be processed in Fall 2020. The report of the Canadian COVID-19 Expert Panel on Testing and Screening Advisory Panel recommended “context specific strategies to improve access to testing and screening in underserved and higher risk communities” [39]. The US Centres for Disease Control (CDC) has a framework for maintaining a functioning public health response to COVID-19 which includes recognizing that testing should be prioritized for symptomatic contacts of positive cases, as well as those who are most likely to be infectious, rather than specimens of asymptomatic contacts and those with lower risk exposures [40].

Our finding that people living in ‘high income’ neighbourhoods had more tests than those in ‘low income’ neighbourhoods, although the latter had higher positivity rates, represents a mismatch between testing needs and COVID-19 risk. To date, Ontario’s testing strategies have been focused primarily on setting out criteria for testing, then disseminating recommendations through media. Throughout the pandemic, criteria around who is ‘eligible’ or ‘recommended’ for SARS-CoV-2 testing have varied not only with the passing of time but also between assessment centres. Within this changing landscape, individuals must understand criteria for testing, know how to access testing and have the time and ability to get to a testing centre. It is not surprising then, that people with financial challenges would have more difficulty accessing testing than those who have more resources.

Given the higher positivity rates among people in ‘lower income’ and ‘higher immigrant’ areas, we question the appropriateness of our province’s testing approach during the study period April–September 2020, given that it did not specifically prioritize high-risk neighbourhoods. We note that our study occurred when test positivity was low, at roughly 2%. Since the start of the third wave in Ontario, COVID-19 test positivity rates have ranged from 10% to over 22% (e.g., Brampton) in some regions of the Greater Toronto Area (GTA) [41]. There are some ‘local’ initiatives, such as ‘pop-up’ testing sites to higher risk neighbourhoods, coordinated through community health centres, Ontario Health Teams [42], clinical teams from local hospitals [43] or through mobile buses [44]. Community engagement and mobilization efforts with volunteers from both the health and social services sectors and local community leaders have tried to ensure that COVID-19 testing is more accessible in Canada [39]. Options for improving accessibility include situating centres in locations that are accessible via public transport or in high-traffic areas (e.g. cultural or religious centres, near grocery stores), and offering evenings/weekend timings to be more accessible for individuals with employment, financial, or childcare-related barriers. Advertising for testing locations and requirements should be available in multiple languages for patients who speak English as a second language, in addition to offering trained medical interpreters [45, 46].

The main limitation of our study is the possibility that people living in lower income neighbourhoods and/or lower immigrant neighbourhoods disproportionately attended assessment centres other than the two included in this study. While this is possible, the direction of the impact would depend on the positivity rate: for example, if people in lower income neighbourhoods who were positive, were more likely to attend other assessment centres, than this would decrease the magnitude of the association between income and COVID-19 positivity in this study. If people living in higher immigration level neighbourhoods who were also positive were more likely to attend other assessment centres, then this would increase the magnitude of the association between immigration status and test positivity here. Our inclusion of assessment centres in neighbourhoods at different income/immigration levels attempted to limit the impact of this, and there is no specific reason to suspect that there were these systematic differences in attendance at different assessment centres. The nearest assessment centre to the NYGH site was 7 km away; there were multiple assessment centres within 1 km of WCH. People may have preferentially attended centres close to their workplace, but that is unlikely to cause substantial systematic differences in positivity rate or income/immigration neighbourhood status. The other possibility is that people living in lower income neighbourhoods were never tested, which would support our finding of a testing/risk mismatch. Assessment centres did not collect race and immigration status data, nor occupation. Despite these being important demographic factors associated with COVID-19, there are no requirements to collect these data in Canada, which has limited our ability to identify those at highest risk and allocate resources, such as testing, appropriately [14]. The results need to be interpreted in the context of changing testing criteria and epidemiology over time (Table 1) [47]. In addition, it is also important to note that income and immigration status may be proxies for other environmental features that may promote infections with COVID-19, such as population density or housing and working conditions. Furthermore, as our immigration-related data was solely based on foreign-born status, future studies may wish to investigate the full dynamics around immigrations, including second-generation or third-generation families. Finally, it is important to note that our data were collected in 2020. Given the rapidly evolving situation of the pandemic, our findings may not reflect the most recent waves and other contexts.

## Conclusions

The COVID-19 pandemic has clearly shown the impact of the social determinants of health on who contracts COVID and who will experience more severe illness. Our findings confirm the increased risk of a positive test among people living in low-income and high immigrant neighbourhoods and also suggest that health inequities extend to who is tested. Moving forward, governments acting on public health guidance must work with local clinical teams, social service organizations, and community leaders and volunteers to eliminate the barriers that marginalized groups face in accessing COVID-19 testing. These study findings could also have implications for other related public health strategies during the COVID-19 pandemic, such as the distribution and access to vaccines.

## Supplementary Information

**Additional file 1: Table S1.** Odds ratios for positive COVID-19 swab test. **Table S2.** Positivity rate of COVID-19 swab test with respect to interaction between immigration terciles and income quintiles.

## Data Availability

The datasets generated and/or analyzed during the current study are not publicly available due to limitations of ethical approval involving the patient data and anonymity but are available from the corresponding author on reasonable request.

## Abbreviations

CDC: Centres for Disease Control
COVID-19: Coronavirus disease 19
GTA: Greater Toronto Area
ICES: Institute for Clinical Evaluative Sciences
NYGH: North York General Hospital
PCR: Polymerase chain reaction
SARS-CoV-2: Severe acute respiratory syndrome-coronavirus − 2
WCH: Women’s College Hospital
