# Supplementary material for: Socioeconomic and immigration status and COVID-19 testing in Toronto, Ontario: retrospective cross-sectional study
Source: BMC Public Health. 2022 May 29;22:1067. doi: 10.1186/s12889-022-13388-2 (PMC9148216; doi:10.1186/s12889-022-13388-2)
Supplement: Supplementary file 1 — Additional file 1: Table S1. Odds ratios for positive COVID-19 swab test. Table S2. Positivity rate of COVID-19 swab test with respect to interaction between immigration terciles and income quintiles. [file 12889_2022_13388_MOESM1_ESM.docx]

**Appendix**

**Table S1: Odds ratios for positive COVID-19 swab test**

| *Type* | *Effect* | *Index group* | *Reference group* | *Odds ratio* | *Lower* | *Upper* | *P-value* |
| --- | --- | --- | --- | --- | --- | --- | --- |
| Crude odds ratio | Income quintiles | 1 | 5(=highest) | 3.52 | 3.041 | 4.085 | <.0001 |
| Crude odds ratio | Income quintiles | 2 | 5(=highest) | 1.94 | 1.644 | 2.287 | <.0001 |
| Crude odds ratio | Income quintiles | 3 | 5(=highest) | 1.46 | 1.219 | 1.750 | <.0001 |
| Crude odds ratio | Income quintiles | 4 | 5(=highest) | 1.44 | 1.200 | 1.718 | <.0001 |
| Crude odds ratio | Immigration terciles | 2 | 1(=lowest) | 1.71 | 1.379 | 2.111 | <.0001 |
| Crude odds ratio | Immigration terciles | 3 | 1(=lowest) | 3.26 | 2.656 | 3.995 | <.0001 |
| Adjusted odds ratio | Income quintiles | 1 | 5(=highest) | 3.38 | 2.661 | 4.290 | <.0001 |
| Adjusted odds ratio | Income quintiles | 2 | 5(=highest) | 1.89 | 1.477 | 2.431 | <.0001 |
| Adjusted odds ratio | Income quintiles | 3 | 5(=highest) | 1.54 | 1.184 | 2.000 | 0.0013 |
| Adjusted odds ratio | Income quintiles | 4 | 5(=highest) | 1.31 | 1.007 | 1.697 | 0.0440 |
| Adjusted odds ratio | Immigration terciles | 2 | 1(=lowest) | 2.15 | 1.540 | 3.016 | <.0001 |
| Adjusted odds ratio | Immigration terciles | 3 | 1(=lowest) | 2.49 | 1.768 | 3.521 | <.0001 |

Table S2: Positivity rate of COVID-19 swab test with respect to interaction between immigration terciles and income quintiles

|  | | *COVID-19 swab test* | | | | P-value† | *Total* |
| --- | --- | --- | --- | --- | --- | --- | --- |
|  |  | *Negative* | | *Positive* | |  |  |
|  |  | *N* | *Row Percent (%)* | *N* | *Row Percent (%)* |  | *N* |
| *Income Quintiles* | *Immigration terciles* | 337 | 98.83% | 4 | 1.17% |  | 341 |
| *1(=lowest)* | *1(=lowest foreign born population)* |  |  |  |  | <0.0001 |  |
|  | *2* | 2566 | 97.34% | 70 | 2.66% |  | 2636 |
|  | *3* | 11969 | 95.86% | 517 | 4.14% |  | 12486 |
| *2* | *1* | 703 | 99.15% | 6 | 0.85% | <0.0001 | 709 |
|  | *2* | 4776 | 98.45% | 75 | 1.55% |  | 4851 |
|  | *3* | 9118 | 97.44% | 240 | 2.56% |  | 9358 |
| *3* | *1* | 892 | 98.89% | 10 | 1.11% | 0.0773 | 902 |
|  | *2* | 6256 | 98.47% | 97 | 1.53% |  | 6353 |
|  | *3* | 6252 | 98.19% | 115 | 1.81% |  | 6367 |
| *4* | *1* | 1659 | 98.93% | 18 | 1.07% | <0.0001 | 1677 |
|  | *2* | 7686 | 98.44% | 122 | 1.56% |  | 7808 |
|  | *3* | 4759 | 98.16% | 89 | 1.84% |  | 4848 |
| *5(=highest)* | *1* | 8262 | 99.18% | 68 | 0.82% | 0.0002 | 8330 |
|  | *2* | 12984 | 98.80% | 158 | 1.20% |  | 13142 |
|  | *3(=highest foreign-born population)* | 2356 | 98.33% | 40 | 1.67% |  | 2396 |
| *Total* | | 80575 | 98.02% | 1629 | 1.98% |  | 82204 |

† P-value assesses the existence of linear trend in increasing positivity rate of COVID-19 swab test with increase in foreign-born population within each income quintile.

N = 82204 in this table (N = 83443 in Tables 1, 2, and 3) because missing data for immigration and income were removed prior to the analysis presented here
